# 

**Published:** 1848-07

**Authors:** 


### INDEX.

PAGE.

A.

Advice on the Management of the Teeth, . . . 94
Am. Soc. Dent. Surgeons, . 97
Anæsthetic Agent, new, 147, 193
Amount of Effort required in the Extraction of Teeth. . 162
Amalgam, . . . 199
Antagonising Instrument, new, 208
Abrasion of the Teeth, , 254
Absorption, Molar and Maxillary, . . . . . 255
Anatomical and Physiological Relations of the Mouth, . 294
Alveoli, Spontaneous Destruction of, . . . . 350

B.

Baltimore College of Dental Surgery, Contributions to Museum of, . . . 110
Blake, W. P., Letter from, . 278
Baltimore College of Dental Surgery, Commencement of, 309

C.

Collateral Branches of Medicine and Surgery, Importance of a Knowledge of, to the Dentist, . . 29, 113, 247
Cardinal Points of Dental Science, &c. . . . . . 93
Case-Book for Dentists, . 95
Carpenter's Physiology, &c., 96
Contributions to the Museum of the Baltimore College of Dent. Surgery, . . . 110

PAGE.

Colburn, G. F. J., Letter from, . . . . . 160
Cone, Dr. C. O., on Extraction of Teeth, . . . 162
Camphor, Effects of, on the Teeth, . . . . . 178
Cleft Palate, and Staphyloraphy, . . . . 184, 279
Correction, . . 192, 208
Chloroform, . . . 193
Colburn, G. F. J., on the Uses of Gutta Percha, . . 258
Circular to Fellows of the A. S. D. S., &c. . . . . 308
Commencement of Baltimore College Dent. Surgery, . 309

D.

Dental Bone, Deposition and Absorption of, . . . 3
Dentistry, Importance of a Knowledge of the Collateral Branches of Medicine and Surgery, in connection with, 29, 113, 247
Dental Physiology and Surgery, a Course of Lectures on, . . . 33, 120, 247
Dentistry, a Treatise on Surgical and Mechanical, . 86
Dental Science and Practice, a Treatise on the Cardinal Points of, . . . . 93
Dentists' Case-Book, Leger and Recorder, . . 95
Dental Surgeons' Eighth Annual Meeting of Amer. Soc'y, 97
Dentition, Third, . . . 106
Desirabode, the Work of, . 109

PAGE.

Dentition, Premature, and Supernumary Teeth, . . . 181

Dental Surgeons, Miss. Valley Association of, . . . . 198

Dental Recorder, New York, 203

Dental Register of the West, 203

Dentition, Fourth, . . . 204

Dental News Letter, . . . 206

Dental Surgery, Circular to the Members of the A. S. of, 308

Dental Surgery, Commencement of Balt. College of, . . 309

Duct of Wharton, Removal of a Foreign Body from, . 312

E.

Effects of Irritation upon the Deposition and Absorption of Dental Bone, . . . 3

Extraction of Teeth, . . . 23

Ether Sulphuric, Inhalation of, . . . . . . 77

Eighth Annual Meeting of the American Society of Dental Surgeons, . . . . 97

England, Subscribers in . . 112

Effects of Camphor on the Teeth, . . . . . 178

Extraction of Teeth, Obstinate Hemorrhage from, . 207

Elements of General Pathology, . . . . . 306

Ether, Discovery of the Application of, . . . . 310

Education, Medico-dental,

Extraction of Teeth, Effort Required in the, . . . . 162

Elliot, W. H., D. D. S., on Absorption of Dental Bone, 3

Eve, Paul F., M. D., Operations on the Jaws, . . 367

F.

Fourth Dentition, . . . 204
Ferguson, William, Esq., F. R. S. E., on Cleft Palate and Staphyloraphy, . 279, 184
Fellows of the American Society, Circular to, . . . 308
Fracture of the Nasal Bones, 383

PAGE.

G.

Gutta Percha and its uses, . 258

H.

Hill, A., D. D. S., on the Teeth with Respect to Voice, 10
Harbert, Samuel C., Practical and Surgical Dentistry, 86
Hamlin, T. B., D. D. S., Advice on the Management of the Teeth, . . . . 94
Harbert, S. C., Letter from, 170
Handy, W. R., M. D., Professor, on Anatomical and Physiological Relations of the Mouth, . . . . 294
Hemorrhage, . . . 207

I.

Inhalation of the Vapor of Sulphuric Ether, . . . . . 77
Importance of Medico-Dental Education, . . . . 109
Irregularity of the Teeth, 253

K.

Kreider, M. Z., M. D., Reports of four cases in Surgery, 352

L.

Lee, J., M. D., on Extraction of Teeth, . . . . 23
Lectures on Dental Physiology and Surgery, 33, 120, 209, 313
Letter from Dr. E. Townsend of Philadelphia, . . 54
Letter from Dr. Thos. S. G. Morton of Boston, . . 56
Lectures by Mr. Tomes, . 109
Letter from Dr. S. Parsons, 159
Letter from G. F. J. Colburn, . . . . 160
Letter from Dr. S. C. Harbert, 170
Letter from Professor Slack, 172
Letter, Dental News, . 206
Letter from W. P. Blake, New York, . . . . . 278
Lining Membrane of the Ear, disease of, . . . . 311
Lee, J., M. D., Neuralgia, 354

PAGE.

M.

Morton, S. G., Dr., Letter from, . . . . 56
Morton, S. G. Dr., Claims to the Discovery of the Inhalation of the Vapor of Sulphuric Ether, . . . . . 77
Mississippi Valley Association of Dental Surgeons, . 198
Molar and Maxillary Absorption, . . . . . 255
Midwifery, Principles and Practice of, . . . . . 306
Management of Irregular Dentition, . . . . . 355
Mitchell, J. B., M. D., on the Management of Irregular Dentition, . . . . 355
M'Calla, John, Thesis, 374

N.

Nævus, . . . . 199
New York Dental Recorder, 203
New Wax Holder, for taking Impressions, . . . 208
New Antagonising, Instrument 208
Neuralgia. . . . . 354

O.

Observations on the Effects of Irritation upon the Deposition and Absorption of Dental Bone, . . . . 3
Observations upon the Importance and Value of a knowledge of the Collateral Branches of Medicine and Surgery to the Dentist, 29, 113
Obstinate Hemorrhage from the Extraction of Teeth, 207
Operations on the Jaws, . 367

P.

Practical Treatise on the Operations of Surgical and Mechanical Dentistry, . 86

Principles of Human Physiology, . . . . . 96

Parsons, S., Dr., Letter, from, 159

Physical Diagnosis, . 162

Parmly, G. W., Dentist, . 311

PAGE.

Pease, Wm. A., a Case of Molar and Maxillary Absorption, 255
Principles and Practice of Midwifery, . . . . . 306
Popular Treatise on the Teeth, 307
Parmly, Eleazar, M. D., Valedictory Address, . . 260
Premature Dentition and Supernumerary Teeth, . 181
Palate, Cleft and Staphylorraphy, . . . 184, 279

R.

Robinson, James, D. D. S., Observations upon the Importance and Value of a Knowledge of the Collateral Branches of Medicine and Surgery, . . 29, 113, 247
Remarks on the Proper Mode of Administering Sulphuric Ether by Inhalation, . 96
Removal of a Foreign Body from the Duct of Wharton, 312
Reports of four Cases in Surgery, . . . . . 352

S.

Shepherd, S. M., D. D. S., a Treatise on the Cardinal Points of Dental Science and Practice, . . . . . 93
Simpson, J. Y., M. D., F. R. S. E., account of a New Anæsthetic Agent as a Substitute for Sulphuric Ether in Surgery, . . . 147
Slack, Professor, Letter from, 172
Stille, Alfred, M. D., Elements of General Pathology, . 306
Smith, G. Mayo, a Popular Treatise on the Teeth, . 307
Subscribers in England, to, . 112
Spontaneous Destruction of the Alveoli, . . . 350
Sheppard, S. M., D. D. S., Spontaneous Destruction of the Alveoli, . . . 350

T.

The Teeth with Respect to the Voice, . . . . . 10

PAGE.
Tomes, John, Esq., Lectures on Dental Physiology and Surgery, . 33, 120, 209
Townsend, E., Dr., Letter from, 54
Treatise on the Cardinal Points of Dental Science and Practice, . . . . 93
Third Dentition, . . 106
Tomes' Lectures, . . 109
Transposition of the Teeth, . 110
Tardiness in the Shedding of the Temporary Teeth, 111
Taft, J., Irregularity of the Teeth, . . . . . 253
Taft, J., Abrasion of the Teeth, 254

PAGE.
Tucker, H. David, M. D., Principles and Practice of Midwifery, . . . . . 306
Thesis on Tic Douloureux, or Neuralgia Faciei, . . 374

V.

Valedictory Address, . 260

W.

Work of Desirabode, . 109
Wharton, the Removal of a foreign body from the Duct of, . . . . . 312

### AGENTS OF THIS JOURNAL.

| | |
|---|---|
| O. P. Laird, M. D. | Columbus, Ga. |
| L. S. Parmly, M. D. | New Orleans. |
| G. McDonald, M. D. | Macon, Ga. |
| W. H. Goddard, | Louisville, Ky. |
| James Taylor, M. D. | Cincinnati, Ohio. |
| A. Baldwin, D. D. S. | Alabama. |
| John Lock D. D. S. | Southport, Wis. |
| E. H. Pebbles, | Lexington, Mo. |
| J. C. Ross, | Nashville, Tenn. |
| J. M. McCurdy, | Philadelphia, Pa |
| M. Z. Kreider, M. D. | Lancaster, Ohio. |
| J. M. Fenn, | Rochester, N. Y. |
| Nicholas Clute, | Louisville, Ky. |
| Benj. Strickland, | Cleveland, Ohio |
| B. B. Brown, M. D. | St. Louis, Mo |
| W. R. Scott, D. D. S. | Raleigh, N. C |
| S. M. Shepherd, | Petersburg, Va |
| John A. Cleveland, | Charleston, S. C |
| W. F. Bason, D. D. S. | N. Carolina. |
| John Lees, | 23 Exchange Arcade, Manchester, England. |

